# Reasons for non-participation in cancer rehabilitation: a scoping literature review

**DOI:** 10.1007/s00520-024-08553-9

**Published:** 2024-05-14

**Authors:** Mikala Erlik, Helle Timm, Anders Thyge Steen Larsen, Morten Quist

**Affiliations:** 1 UCSF—Centre for Health Research, Copenhagen University Hospital—Rigshospitalet, Copenhagen, Denmark; 2 Department of Public Health, University of Copenhagen, Copenhagen N, Denmark; 3 National Institute of Public Health, University of Southern Denmark, Copenhagen, Denmark; 4 Faculty of Health Science, University of the Faroe Islands, Tórshavn, Faroe Islands

**Keywords:** Rehabilitation, Patient participation, Refusal to participate, Health inequities, Social support, Patient acceptance of health care

## Abstract

**Background:**

Rehabilitation plays an important role in addressing the many challenges of living with cancer, but a large proportion of people with cancer do not participate in available cancer rehabilitation. Hence, reasons for non-participation in cancer rehabilitation need to be explored.

**Objective:**

The present study undertakes a scoping review of research examining reasons for non-participation in cancer rehabilitation among people with cancer.

**Design:**

A systematic search was conducted in PubMed, Scopus and CINAHL for articles published until July 2023. Included studies were hand searched for relevant references and citations.

**Eligibility criteria:**

Method: Studies with qualitative, quantitative or mixed-method design. Population: Studies targeting adults (> 18) living with cancer, not participating in rehabilitation. Program type: The review included all studies defining program as rehabilitation but excluded clinical trials. Outcome: Studies examining reasons for non-participation in available rehabilitation.

**Data extraction:**

The extracted data included author(s)/year of publication, aim, population, information, rehabilitation type and main reasons for non-participation.

**Results:**

A total of nine studies were included (*n* = 3 quantitative, *n* = 2 qualitative, *n* = 4 mixed methods). Reasons for non-participation included physical, psychosocial and practical aspects. The main reason across studies was ‘no need for public support’ related to receiving sufficient support from family and friends. All studies focused on individual reasons, and structural conditions were rarely present.

**Conclusion:**

Research within this field is sparse. Future research should explore how individual reasons for non-participation relate to structural conditions, especially among people in socially disadvantaged positions living with cancer.

**Supplementary Information:**

The online version contains supplementary material available at 10.1007/s00520-024-08553-9.

## Introduction

Cancer is a leading cause of death worldwide, and incidence of cancer is growing globally [1]. Early detection and advances in treatment have improved the survival outcomes for many cancers, but cancer remains a common cause of disability and psychosocial distress, often requiring public support [2, 3]. In all parts of the world, the diagnosis of cancer is experienced as traumatic, and the disease often affects all aspects of the diagnosed individual’s life [4, 5]. People with cancer experience different physical, cognitive and emotional symptoms, due to disease progression and invasive treatment [3, 6–9]. As the number of patients treated for and living with cancer continues to rise, these challenges are becoming more evident [10].

Cancer-specific rehabilitation includes a broad range of activities aimed at improving the management of cancer symptoms. Rehabilitation programs have been reported to improve function, reduce symptom burden and enhance the well-being of people experiencing disabilities related to their cancer diagnosis [9, 11]. Considering the numerous complex symptoms associated with cancer, rehabilitation is increasingly seen as an essential component of comprehensive cancer care.

Despite the perceived benefits, many people with advanced cancer do not participate in rehabilitation [12]. Within the Danish healthcare system, this is particularly true for people with low education and income, who are highly underrepresented in rehabilitation, despite reporting several unmet needs and cancer-related challenges [3, 13].

An increasing body of literature examines the facilitators and barriers to cancer survivors* participation in exercise interventions and clinical trials [14, 15]. One review identified barriers, facilitators and preferences associated with cancer patients’ participation in hospital-based exercise programs but did not include a wider range of psychosocial and practical rehabilitation programs [16]. This poses a challenge as a significant number of people with cancer experience severe emotional disturbance and limitations in daily activities [9, 17]. In addition, assessments of non-participation in clinical trials may not be applicable to rehabilitation [14]; people who take part in clinical trials are often required to participate in documentation and testing [15], which may lead to different reasons for non-participation than those associated with publicly available rehabilitation.

To the best of our knowledge, no review has been made on patients’ reasons for not participating in cancer rehabilitation. A scoping review is necessary to provide an overview of existing knowledge and to direct further research efforts.

The aim of this scoping review is to systematically map research investigating the reasons why people with cancer do not participate in available rehabilitation. The objectives are to [1] identify the extent of research examining reasons for non-participation in cancer rehabilitation, (2) to compare and synthetize identified reasons and (3) to pinpoint gaps and areas for further research.

## Methods

Scoping reviews are appropriate for mapping the existing evidence in a broad thematic area as rehabilitation [17]. Methodologically we used the framework by Arksey and O’Malley’s, supported by Preferred Reporting Items for Systematic Reviews (PRISMA-ScR) to ensure a transparent and systematic approach [17, 18]*.*

A preliminary search of PubMED,^1^ the Cochrane Database of Systematic Reviews^2^ and JBI Evidence Synthesis^3^ revealed that no studies have synthesized existing research on the reasons why people with cancer do not participate in rehabilitation.

### Term definitions

Cancer rehabilitation is the contextual field of research framing the scope. It is a complex field because it is inadequately defined and understood by both healthcare professionals and researchers [19]. The lack of comprehensive and shared definitions makes it difficult to determine which types of supportive care should be considered cancer rehabilitation, and which should not.

The World Health Organization (WHO) defines rehabilitation as programs ‘[d]esigned to optimize functioning and reduce disability in individuals with health condition’ [20]. This broad definition provides several possibilities for the organization and implementation of rehabilitation, which can be challenging to classify, compare and synthesize in a scientific review. Due to the broad definition, there has been a tendency to index research within the concept ‘rehabilitation is what rehabilitation professionals do’ [19]. Given this tendency, we used ‘rehabilitation’ as an overall search term, including all studies that defined their research area as cancer rehabilitation led by professionals.

Researchers suggest that cancer-specific rehabilitation should be integrated at all stages of the cancer trajectory [21]. The most influential classification system divides cancer rehabilitation into following four stages: preventive, restorative, supportive and palliative rehabilitation [22]. All four stages were included in our definition of cancer rehabilitation. This broad definition gave us a better opportunity to review the collected research on reasons for non-participation in all stages of cancer rehabilitation, including a wide range of intervention areas.

Throughout the review we use the terminology ‘rehabilitation program’ to define the activities, initiatives and services provided in the context of rehabilitation. We found this terminology more neutral than ‘rehabilitation intervention’ or ‘rehabilitation service’, because ‘intervention’ is often perceived as action-oriented, whereas ‘service’ can make the participants appear as passive recipients.

### Search strategy [23]

A systematic literature search was conducted in collaboration with an experienced librarian (ATSL). An initial limited search was undertaken to identify studies in the field, using index terms in the titles and abstracts of relevant studies. Keywords used were: ‘cancer’, ‘non-participation’ and ‘rehabilitation’. Synonyms for each term were identified through the initial limited search.

Three electronic databases (PubMed, Scopus and CINAHL) were searched to identify all published studies within medical, sociological and nursing literature. The last search was performed on 14th July 2023. Hand searches for peer-reviewed studies were conducted within the following organizational webpages: ‘World Rehabilitation Alliance’[24] ‘Rehabilitation international’ [25] and ‘Cancer.Net’ [26] using the terms ‘Rehabilitation’ and ‘non-participation’ (*including synonyms). The final search strategy is shown in Appendix 1.

### Eligibility criteria

The modified PICo framework for qualitative reviews was used to develop eligibility criteria [27]. Qualitative research provides insights into participants experience and fits the reviews orientation toward patients experienced reasons for non-participation. Peer-reviewed journal articles were eligible for inclusion if they were written in English or Danish, included adults (> 18) with cancer and reported reasons for non-participation in rehabilitation. Quantitative, qualitative and mixed methods studies were all included to consider different arguments and perspectives on the non-participation.

Studies were excluded if they focused on cost-effectiveness, prevention or the effects of rehabilitation, and if they dealt with health professionals, carers or relatives rather than people with cancer. Studies addressing rehabilitation programs that cost money were excluded, as these might point to structural reasons that do not explain non-participation in universal health care systems. Grey literature, abstracts and protocols were excluded as they did not allow for transparent descriptions of methodology, population and findings (Table 1).

**Table 1:** Eligibility criteria used to screen literature in scoping review, framed by PICo

PICo—Eligibility criteria	
***P****opulation*	**> 18 years old** **Living with cancer (after potential surgery)** **Non-participan**t **Exclude:** **Participants in ‘risk’ of cancer** **Health professionals’ or relatives’ perspective**s
***I****nterest*	Explores reasons for non-participation in rehabilitation **Exclude:** Studies exploring participants’ experiences without addressing reasons for non-participation Studies exploring different adherences to programs
***Co****ntext*	Cancer rehabilitation: a freely available program addressing physical, psychological and/or social needs **Exclude:** Programs focusing on general exercise behaviour Programs designed as clinical trials Programs not freely available

### Study selection

Search results were screened using a two-stage process. In the first stage, two reviewers (ME and MQ) independently screened titles and abstracts against the eligibility criteria. Disagreements were resolved by discussion with the third reviewer (HT). Based on discussion, ‘post hoc’ exclusion criteria were developed and applied [17]. The ‘post hoc’ basis for exclusion was studies investigating reasons for non-participation in interventions designed as clinical trials, even if they were described as ‘rehabilitation trials’ [28]. Studies that examined participants’ or health professionals’ perceived reasons for non-participation were only included if they also addressed non-participant reasons. In these cases, data extraction and discussion focused only on data relating to non-participants.

In the second stage, the full text was assessed against the inclusion criteria by the two reviewers (ME and MQ). Any disagreements were resolved by discussion. For all studies included after second screening, reference lists and related articles were screened. First authors were searched using Web of Sciences ‘cited reference search’. Relevant studies not already included were identified for full review.

Several studies focused on drop-outs as a category of non-participation. Drop-outs were defined as people who had attended rehabilitation programs a limited number of times. After full screening we decided to include a few studies exploring drop-outs reasons. Of these, all but one focused on both drop-outs and people never attending (see Table 2). These studies showed that the two groups reported similar reasons, only people who never attended experienced the reasons as more severe [29–31]. In the absence of studies on people who never attended, studies that include drop-outs provided a useful addition to the collective understanding of reasons for non-participation.

**Table 2:** Calculation of non-participants definitions among studies included in the scope. X indicates category of participants. Number of participants in the category is specified in brackets

No registration or referral	Cancelled registration or referral before program start (*n*)	Failed to attend (*n*)	Dropped out: attendance of < 50% (*n*)	
X (368)				Eakin and Strycker (2001)
X (95)				Plass and Koch (2001)
X (26)			X (87)	Ussher et al. (2008)
		X (16)	X(17)	Fitzpatrick and Remmer (2011)
X (221)				Clover et al. (2015)
X (35)				Handberg et al. (2015)
X (212)				Cheville et al. (2017)
	X (9)	X (5)	X (6)	Hardcastle er al. (2018)
			X (6)	Toivonen et al. (2020)
***N* = 957**	***N* = 9**	***N* = 21**	***N* = 116**	**Total**

### Data extraction

Included studies were read carefully to get an overall impression. Studies were divided among ME, MQ and HT. Two reviewers independently conducted data extraction for each study, and key information was extracted and summarized in Appendix 2. The identification of ‘reasons’ was based on direct quotations from the studies based on the perspectives of the participants. Only reasons reported by non-participants with cancer were extracted and added to the data chart. Extracted reasons were organized into groups, to provide an overall perspective on themes emerging from the literature, pertaining to the research question [17]. Major themes were further developed by merging overlapping themes across the included studies. Extracted data were discussed during team meetings, and data chart were updated in an iterative process.

## Results

### Characteristics of sources evidence

A total of nine peer-reviewed studies published between 2001 and 2020 were included in the review [29–37]. Included sources were authored in five different countries: USA, Canada, Australia, Germany and Denmark. The search yielded 881 potential studies. Of these, 856 were excluded during title/abstract screening. The main reason for exclusion was wrong field, including clinical trials, feasibility studies and general physical activity. A total of 156 studies were excluded because they focused on clinical trials, while 52 were excluded because they addressed physical activity or exercise behaviour in general, rather than rehabilitation programs led by professionals.

**Fig. 1:**
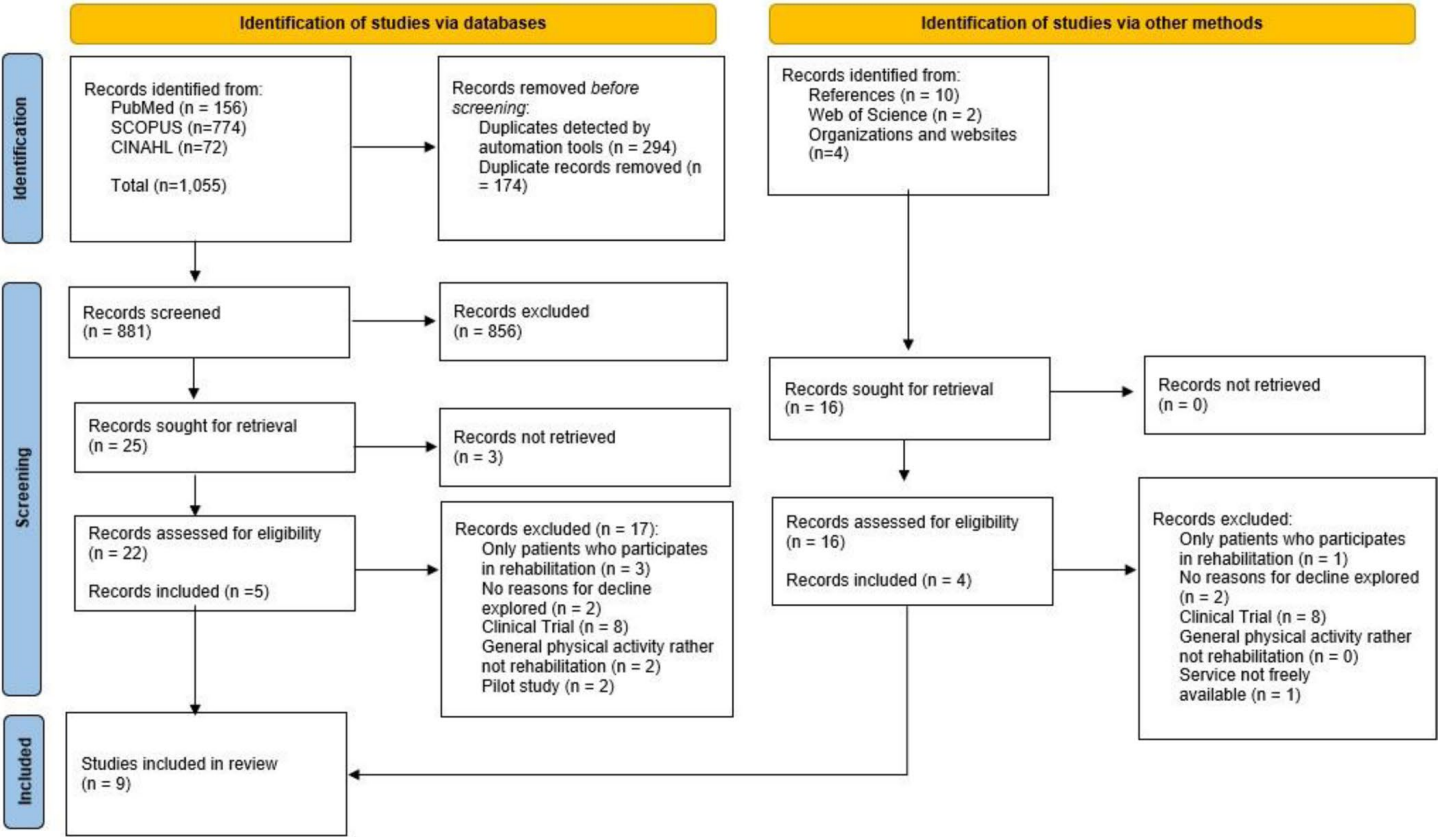
Selection of studies included in the scope

A total of 22 studies were full text screened, and five were included in the final scope. Reference searches and hand-searches of relevant organizations’ websites resulted in full text screening of 16 sources, of which four studies were included. A PRISMA flowchart (Fig. 1) outlines the selection process.

Evidence on reasons for non-participation was all based on participants’ own experiences and explanations. The most common method was interviews (*n* = 5) [29–32, 34], followed by questionnaires (*n* = 3) [35–37]. One study used ethnographic methods to explore how patients’ perceptions and ‘meanings’ influence reasons for non-participation [33].

Included studies represented different types of programs, all of which fell within the definition of freely available cancer rehabilitation. The programs included psychosocial support groups (*n* = 5)[29, 30, 35–37], exercise programs (*n* = 3) [29, 31, 32], mindfulness-based cancer recovery (*n* = 1) [31], cooking classes (*n* = 1) [29] and art therapy (*n* = 1) [29]. The programs were all group-based and delivered in a public setting, such as a health centre or a clinic. Three studies focused on non-participation in general rehabilitation programs [33, 34, 37].

Included studies represented a population of 1105 adults with different types of cancer: lung (*n* = 230), prostate (*n* = 194), breast (*n* = 184), bowel/colon/rectum (*n* = 85), lymphoma (*n* = 17), head/neck (*n* = 11), anus (*n* = 4), urinary (*n* = 4), gynaecologic (*n* = 3), brain (*n* = 2), ovarian (*n* = 1), pancreas (*n* = 1), melanoma (*n* = 1) and tongue (*n* = 1). A total of 364 were either undefined or categorized as ‘other’.

The population of interest was further characterized by different types of non-participants. Identified types were people not referred to or registered in available rehabilitation interventions, people who have cancelled registration, who failed to attend or people who dropped out of rehabilitation. Drop-out was defined as having an attendance of less than 50% of the full programs [29, 30, 32].

Six studies accounted for socioeconomic data on population (20,21,22,24,26,27). Data were of different values, but generally showed a preponderance of highly educated, employed, white patients living with a partner.

### Reasons for non-participation

A total of eight reasons were identified across the included studies: Cancer symptoms, side effect of treatment, preference for other activities, no motivation, low self-esteem, no need for public support, do not relate to position or participants, conflict with other plans, transport issues, bad timing and lack of referral.

The reasons were grouped into three major themes: (1) physical, (2) psychosocial and (3) practical. Table 4 provides an overview of the reasons found in the studies linked to the themes.

The most common reason was the psychosocial, followed by the practical and physical. All included studies identified psychosocial reasons, and six identified psychosocial reasons as the main barriers to participation [30, 33–37] (Table 3).

**Table 3:** Summary of major and minor themes on reasons for non-participation identified across the nine studies. Minor themes list quotes and highlights from the studies, which are further descripted in the data chart (Appendix 2)

Major themes	Minor themes	References
**Physical**	**Symptoms (A)** • Cancer-related pain or fatigue	Ussher et al. (2008); Fitzpatrick and Remmer (2011)
	**Treatment (B)** • Fatigue from chemotherapy	Fitzpatrick and Remmer (2011); Cheville et al. (2017); Toivonen et al. (2020)
**Psychosocial**	**Preference for other activities (C)** • Preferring to talk with someone else • Rehabilitation not fitting preferences • Do not fit personal perceived needs and competences	Plass and Koch (2001) Fitzpatrick and Remmer (2011); Handberg et al. (2015); Hardcastle et al. (2018) Fitzpatrick and Remmer (2011); Hardcastle et al. (2018)
	**No motivation (D)** • Feeling too lazy or lacking self-discipline	Hardcastle et al. (2018); Toivonen et al. (2020)
	**Low self-esteem (E)** • Afraid to do rehabilitation activity wrong or look stupid	Hardcastle et al. (2018); Toivonen et al. (2020)
	**No need for public support (F)** • Feeling sufficient supported by family, friends, religion or hospital/treatment • Not in need of assistance/able to self-manage • It is time to move on and support is not relevant • Previously received support which they found unhelpful	Plass and Koch. (2001); Ussher et al. (2008); Clover et al. (2015); Cheville et al. (2017); Toivonen et al. (2020); Eakin and Strycker (2001) Plass and Koch (2001); Clover et al. (2015); Cheville et al. (2017) Ussher et al. (2008); Handberg et al. (2015) Cheville et al. (2017)
	**Do not relate to participants (G)** • Unprofessional or dominant leader/facilitator • It is too depressing to participate • Not self-identifying as a person with cancer • Not identifying with the practice (of ‘opening up’, showing feelings, being extrovert) • Do not relate to people attending the service	Ussher et al. (2008) Ussher et al. (2008) Ussher et al. (2008) Ussher et al. (2008); Handberg et al. (2015) Ussher et al. (2008)
**Practical**	**Conflict with other plans (H)** • Clash with other commitments (work schedule, childcare, medical appointments, treatment)	Ussher et al. (2008); Fitzpatrick and Remmer (2011); Cheville et al. (2017); Hardcastle et al. (2018); Toivonen et al. (2020)
	**Transportation issues (I)** • Transport to time-consuming or difficult • Parking issues	Ussher et al. (2008); Cheville et al. (2017); Hardcastle et al. (2018) Fitzpatrick and Remmer (2011)
	**Bad timing (J)** • Not the right time • Waiting to recover from treatment or test response	Ussher et al. (2008) Cheville et al. (2017)
	Lack of referral** (K)** • Not recall receiving advice or referral related to rehabilitation	Hardcastle et al. (2018); Eakin and Strycker (2001)

### Physical reasons

The included studies found that cancer symptoms and treatment side effects were the most common physical reason for non-participation [29, 31, 34]. In a mixed methods study by Cheville et al., 17 out of 311 people reported that they were waiting to recover from chemotherapy or radiation, 20 did not believe rehabilitation would be beneficial due to the status of their cancer and 29 found rehabilitation too burdensome because they were in ‘too much pain’ [34].

Fatigue and pain were the most reported side effects, outweighing the physical reason. In the qualitative studies, people with cancer were quoted saying ‘I was unable to go because of pain and fatigue’ [29]. Further: ‘When you are dealing with the feeling of total lack of energy, feeling like your life was revolving around when you get to take your anti-nausea meds next, your life gets very slow, and your life gets very focused upon managing the symptoms of the chemo (…)’ [31].

Across the included studies, two reported treatment-related symptoms as the first and most common reason for non-participation overall [29, 31]. Two studies had a limited focus on treatment or disease-related reasons but mentioned them briefly [30, 32]. On the other hand, two studies using multiple-choice questionnaires did not mention physical reasons at all [35, 36].

### Psychosocial reasons

Psychosocial reasons were the most dominant across the different study methods, populations and types of rehabilitation. Most studies noted that many non-participants reported no need for public support [30, 31, 33–37]. Some studies linked ‘no need for rehabilitation’ to self-management (*n* = 3) [34, 36, 37]. For example, Clover et al. used quantitative methods to include a larger population group not attending cancer rehabilitation and found that 46% (*n* = 99) of people declined rehabilitation programs because they preferred to manage life with cancer by themselves. This reason was particularly prevalent in studies with a predominance of participants not enrolled or referred to rehabilitation (Table 2). However, receiving sufficient support elsewhere was more dominant across all studies, with ‘no need for rehabilitation’ as the most common reason (*n* = 4) [30, 35–37]. A questionnaire conducted by Plass and Koch found that 75 of 94 people reported adequate support from family, 54 of 94 from friends and 43 of 94 preferred to talk to a doctor. People with cancer were moreover quoted saying: ‘When I’m feeling down my family supports me’(30, 35–37). Suggesting that family are the most common reason for not participating in public support programs, but the studies do not explore how or on what parameters.

The second most reported psychosocial reason was ‘preference for other activities’ [29, 32, 33]. People with cancer, who preferred other activities, did not identify themselves with someone in need of support [33], did not relate to people attending rehabilitation [30] or felt that the program did not fit their competences or daily structure [29, 32].

### Practical reasons

Time and transport were a major practical barrier for participation. People with cancer reported that rehabilitation conflicted with other commitments and appointments [29–32, 34]. Transport issues were the most reported practical reason [30, 32, 34]. People with cancer were quoted saying: ‘It [transportation] takes quite a bit out of the day so I’m not sure I would do that’ [32] and ‘You should make a parking available for Centre participants’[29].

On contrast, some included studies suggested that the practical reasons played a minor role. In the study by Cheville et al. only 12 of 311 people mentioned transport as a barrier to participation [34]. Similarly, the study by Eakin et al. found that only 7% of respondents cited ‘The location is inconvenient’, 4% cited ‘I don’t have transportations’ and 4% cited ‘The service is offered at inconvenient times’ as reasons for non-participation [35] (Table 4)

**Table 4:** Overview of themes highlighted in each of the nine studies

Physiological		Psychosocial					Practical			
(A) Symptoms	(B) Treatment	(C) Preference for other activities	(D) No motivation	(E) Low self-esteem	(F) No need for public support	(G) Do not relate to participants	(H) Conflict with other plans	(I) Transportation issues	(J) Bad timing	(J) Bad timing
					+ +					+ +
		+			+ +					
+					+	+ +	+ +	+ +	+ +	
+ +	+ +	+					+	+		
		+			+ +					
		+ +			+	+ +				
+	+				+ +		+	+	+	
	+	+ +	+	+			+	+	+	+
	+	+ +	+	+			+ +			
+ = 4	+ = 5	+ = 9	+ = 2	+ = 2	+ = 10	+ = 4	+ = 7	+ = 5	+ = 4	+ = 3

( +) Reason highlighted in study. (+ +) Reason described as being of significant value.

## Discussion

The aim of the review was to assess the extent of existing research and synthesize existing knowledge about the reasons why people with cancer do not participate in rehabilitation. In this section, the findings are discussed in relation to research gaps to direct future research.

A strength of our review is that it adopted a rigorous systematic search strategy, following the PRISMA-ScR guidelines. Nine studies met the inclusion criteria, but they varied in terms of method, population and type of rehabilitation. Looking at the included studies, they contained similar reasons across methodical approach, date, country, rehabilitation service and cancer population. The reasons given by people with cancer were categorized as either physical, psychosocial or practical.

The most dominate reason was ‘no need for public support’, explained by having sufficient support elsewhere. In this context, support from family and friends appeared to be the main reason for non-participation, but studies lack further exploration of how peer support influences rehabilitative support in cancer, e.g. when and how peer support acts as prevention or as a resource for the psychological, social and functional effects of cancer [32].

The fact that all included literature finds similar physical, psychosocial and practical reasons, regardless of method, program type and country, suggests that the major themes interact to determine non-participation in rehabilitation. Toivonen et al. find that it is the interaction between factors that prevent participation, rather than single causal factors [31]. Studies included in the review show how ‘preferences’, thematized as a psychosocial reason, interrelate with both physical and practical elements. For example,’[I prefer] some mild simple exercise’ [32] could refer to physical condition or ability, while ‘[it would be] better if someone could come to your house’ [32] refers to a preference that could both be related to both a psychosocial need for a safe environment and practical transport problems. Future studies should explore how factors interrelate and influence each other across different groups of non-participants.

It is unanticipated that ‘no need for public support’ is the most dominant reason for non-participation. Research concludes that people with low socioeconomic status are often the ones who do not participate in rehabilitation programs and trials [13, 38] despite experiencing multiple cancer-related problems [39, 40]. The reason why public support is not considered necessary may be due to different perceptions of support or lack of trust in the public system, but concrete reasons related to socioeconomic position are not explored in any of the included studies. A growing research field exploring inequalities in cancer suggests that people with socioeconomic vulnerability are poorly represented in research [13, 38, 41]. The results of this scoping review imply that this is also the case for research exploring reasons for non-participation in cancer rehabilitation.

A limitation of our review is the search strategy, which was limited to three databases. However, due to the extent of the citation and reference search, it is likely that we identified existing relevant results. The databases and sources are further considered appropriate as a wide range of studies were identified, especially in the light of similar reviews finding limited studies on cancer rehabilitation [42]. Our selection and exclusion process showed that the reasons for non-participation in clinical trials have been more thoroughly researched. By comparing our findings with studies in this area, we were able to validate our results and extend our understanding of non-participation. A systematic review examining recruitment rates to exercise trials among cancer survivors found that barriers to participation were predominantly patient-centric, with transport issues, disinterest, time and commitment being the most common barriers [43]. In addition, a recent scoping review of physical activity participation among people with cancer identified both physical, psychosocial, cultural, economic and environmental issues as factors influencing participation [16]. The mentioned findings correspond well with the major themes identified in this review.

In line with the scoping review genre, we did not assess potential bias or quality of evidence [17], but during the screening and extraction process it became clear that the included studies had varying levels of methodological bias and rigour. For example, in the study by Cheville et al., several people reported not attending due to cancer or treatment-related side effects, but physical reasons were not mentioned at all in the discussion or conclusion. Instead, the authors emphasized psychosocial reasons, stating that ‘[t]he most prevalent barrier to rehabilitation was the perception that it is not needed’ [34]. Similarly, the two studies using predominantly qualitative methods were the only ones to highlight social position and identification as a central factor [30, 33]. On the other hand, a multiple-choice questionnaire did not elicit responses related to either physical factors or social positions [36]. Thus, conclusions and perspectives were influenced by the research objective, methodology and area of interest, making it difficult to compare the studies.

Comparison was further complicated by varied definitions of rehabilitation and non-participation, as nearly half of the included studies were identified by methods other than database searches (Fig. 1). Like others, we have found that there is a need for a shared definition of cancer rehabilitation to conduct a comprehensive systematic review in this area [19]. In this scoping review, the most frequently explored rehabilitation types were exercise or psychological support groups. Other areas of cancer rehabilitation were sparsely covered, highlighting the need for more knowledge about a wider range of rehabilitative programs. In addition, there is a need for more awareness of cancer-related issues to cover all potential symptoms occurring at all stages of the cancer trajectory [30, 33]. Given the varying definitions and methodological quality, it is doubtful whether the existing knowledge is sufficient.

Following our orientation on people with cancers perspectives, ‘reason’ has been a key term structuring the search strategy, eligible criteria as well as the data extraction. This is a limitation, which may have led to an overweighting of micro-level studies that explore individual explanations for non-participation. This may also have influenced the overall emphasis on psychosocial reasons throughout the review. A broader focus on ‘barriers’ or ‘mechanisms’ could include studies that explore the issue at a more structural or institutional level. Research on barriers to participation in clinical trials suggest an “enormous need to address structural and clinical barriers” [14] and point to the fact that structural barriers are the reason why three out of four people with cancer do not participate in clinical trials. Future studies should include both individual and structural reasons for non-participation.

### Clinical implications

A more comprehensive understanding of reasons for non-participation may be beneficial for clinicians and policymakers developing supportive care in cancer. Our findings should be considered in the screening process and when discussing referral to supportive care with patients.

## Conclusion

This scoping review shows that research into the reasons for non-participation in rehabilitation among people with cancer is sparse. From the body of the including studies, it was possible to discern some general physical, psychosocial and practical reasons for non-participation.

The findings highlight the need for further research into the reasons for non-participation in cancer rehabilitation. Emphasis should be placed on how reasons interrelate and reinforce each other at both individual and structural level. People with cancer from low socioeconomic backgrounds are underrepresented in both research and rehabilitation. We recommend further research targeting this specific group as means to explore how social position is related to supportive cancer care and cancer rehabilitation.

### Supplementary Information

Below is the link to the electronic supplementary material.

Supplementary file1 (DOCX 26 KB)

Supplementary file2 (DOCX 33 KB)

## Data Availability

The authors confirm that the data supporting the findings of this study are available within the article or its supplementary materials.

## Footnotes

1 Using search terms: Cancer AND Nonparticipation AND Rehabilitation: 50 results was found and 0 reviews.

2 Using search terms: Cancer AND Nonparticipation AND Rehabilitation: 11 trials was found and 0 Cochrane Reviews.

3 Using search terms: Cancer AND Nonparticipation AND Rehabilitation: 0 results was found.
