# Supplementary material for: Reasons for non-participation in cancer rehabilitation: a scoping literature review
Source: Support Care Cancer. 2024 May 14;32(6):346. doi: 10.1007/s00520-024-08553-9 (PMC11093823; doi:10.1007/s00520-024-08553-9)
Supplement: Supplementary file 1 — Supplementary file1 (DOCX 26 KB) [file 520_2024_8553_MOESM1_ESM.docx]

**Appendix I: Search Strategy**:

**Q:** Research exploring factors influencing nonparticipation in public rehabilitation programs, among people living with cancer.

No limits initially on dates.

**Identification of studies via databases**

**PubMed.** Date of search 17/07/23

| **#** | **Searches** | **Results** |
| --- | --- | --- |
| 1 | neoplasm [mh] OR cancer [tiab] OR oncolog*[tiab] | [4,493,774](https://pubmed.ncbi.nlm.nih.gov/?term=neoplasm+%5Bmh%5D+OR+cancer+%5Btiab%5D+OR+oncolog%2A%5Btiab%5D&sort=) |
| 2 | nonparticipation[tiab] OR nonparticipants[tiab] OR "withdrawl"[tiab] OR dropout[tiab] OR refusal[tiab] OR decline group[tiab] OR participation barriers[tiab] | [32,425](https://pubmed.ncbi.nlm.nih.gov/?term=nonparticipation%5Btiab%5D+OR+nonparticipants%5Btiab%5D+OR+%22withdrawl%22%5Btiab%5D+OR+dropout%5Btiab%5D+OR+refusal%5Btiab%5D+OR+decline+group%5Btiab%5D+OR+participation+barriers%5Btiab%5D&sort=) |
| 3 | rehabilitation[mh] OR rehabilitation[tiab] OR recovery[tiab] | [1,017,108](https://pubmed.ncbi.nlm.nih.gov/?term=rehabilitation%5Bmh%5D+OR+rehabilitation%5Btiab%5D+OR+recovery%5Btiab%5D&sort=) |
| 4 | #1 AND #2 AND #3 | [159](https://pubmed.ncbi.nlm.nih.gov/?term=%231+AND+%232+AND+%233&sort=) |
|  | **Results exported to Rayyan and Zotero** | **Total 156** |

**Scopus.** Date of search 17/07/23

| **#** | **Searches** | **Results** |
| --- | --- | --- |
| 1 | ( TITLE-ABS-KEY ( "cancer" ) OR TITLE-ABS-KEY ( oncolog* ) OR ALL ( neoplasms ) ) | **5,030,132** |
| 2 | ( TITLE-ABS-KEY ( nonparticipation ) OR TITLE-ABS-KEY ( nonparticipants ) OR TITLE-ABS-KEY ( "withdrawl" ) OR TITLE-ABS-KEY ( dropout ) OR TITLE-ABS-KEY ( refusal ) OR TITLE-ABS-KEY ( decline AND group ) OR TITLE-ABS-KEY ( participation AND barriers ) ) | 232,740 |
| 3 | ( TITLE-ABS-KEY ( rehabilitation ) OR TITLE-ABS-KEY ( recovery ) OR TITLE-ABS-KEY ( activities AND of AND daily AND living ) ) | **1,835,218** |
| 4 | #1 AND #2 AND #3 | **774** |
|  | **Results exported to Rayyan and Zotero** | **Total 774** |

**CINAHL.** Date of search 17/07/23

| # | Searches | Results |
| --- | --- | --- |
| 1 | (MH "Neoplasms+") OR (cancer) OR (oncolog*) | **848.280** |
| 2 | (nonparticipation) OR (nonparticipants) OR ("withdrawl") OR (dropout) OR (refusal) OR (decline group) OR (participation barrier*) | **23,345** |
| 3 | (MH “Rehabilitation+”) OR (Rehabilitation) OR (recovery) | **558,975** |
| 4 | S1 and S2 and S3 | **72** |
|  | **Results exported to Rayyan and Zotero** | **Total 72** |

**Identification of studies via other methods**

**Web of Science**

|  | **Searches** | **Results** |
| --- | --- | --- |
|  | *Author search*: Le Rouzic, Oliver  Search within results: rehabilitation  0 articles eligible for inclusion | 14 |
|  | *Author search*: Cheville, Andrea L  Search within results: rehabilitation  Only 1 article eligible for inclusion – already included. *Relevant article search:* (“How Receptive Are Patients With Late Stage Cancer to Rehabilitation Services and What Are the Sources of Their Resistance?”)34 citations and 41 cited references2 articles were eligible for inclusion | 93 |
|  | *Author search*: Flores, Ann Marie  Search within results: rehabilitation  Only 1 article eligible for inclusion – already included. (see above) | 16 |
|  | *Author search*: Handberg Charlotte  Search within results: rehabilitation  Only 1 article eligible for inclusion – already included. *Relevant article search:* (“Understanding male cancer patients’ barriers to participating in cancer rehabilitation”)32 citations and 58 cited references 0 articles eligible for inclusion | 49 |
|  | **New results exported to Rayyan and Zotero** | **Total 2** |

**Citation search**

|  | **Searches** | **Results** |
| --- | --- | --- |
|  | All studies assed for eligibility were hand-searched in ‘references’, ‘cited by’ and ‘similar articles’ categories |  |
|  | **Results exported to Rayyan and Zotero** | **Total 10** |

**Organizations and websites**

|  | **Searches** | **Results** |
| --- | --- | --- |
|  | World Rehabilitation Alliance (<https://www.who.int/initiatives/world-rehabilitation-alliance>) | 3 |
|  | Rehabilitation International (<https://www.riglobal.org/about/>) | 1 |
|  | Cancer.Net (<https://www.cancer.net/search/site/rehabilitation%2520decline>)  Search terms: Rehabilitation AND refusal OR decline OR nonparticipation  0 articles eligible for inclusion | 13 |
|  | **Results exported to Rayyan and Zotero** | **Total 4** |
|  |  |  |
