# Supplementary material for: Reasons for non-participation in cancer rehabilitation: a scoping literature review
Source: Support Care Cancer. 2024 May 14;32(6):346. doi: 10.1007/s00520-024-08553-9 (PMC11093823; doi:10.1007/s00520-024-08553-9)
Supplement: Supplementary file 2 — Supplementary file2 (DOCX 33 KB) [file 520_2024_8553_MOESM2_ESM.docx]

| Author | Aim | Methods | Population | Rehabilitation-type | Reasons for nonparticipation |
| --- | --- | --- | --- | --- | --- |
| Eakin & Strycker (2001) | Aims to explore cancer patients’ awareness and use – as well as obstacles to use – of Health maintenance organization (HMO) and community based psychosocial programs designed for cancer patients. | Quantitative  Randomized design- survey  Mailed survey including a list of 13 barriers to use cancer support. | 501 patients diagnosed between 9 and 24 months prior to the study were randomly selected from HMO tumor registry  368 returned surveys  HMO: Health maintenance organization is a medical insurance group that provides health programs for a fixed annual fee. | HMO-and community based psychosocial support programs designed for cancer patients  Free, HMO’s Cancer Counseling Center offers free individual and group counseling programs for all cancer patients and their families  United states | 1 % answered: I’m too sick  4 % answers: The programs are offered at inconvenient times, I don’t have transportation, my time and energy are focused on my treatment, counseling can be more upsetting than helpful, counselling isn’t relevant to dealing with cancer.  6% answered: I’m uncomfortable seeking counseling.  7% answers: The location is inconvenient, I’m to busy  Most common reasons:  **I don’t know enough about it** (9%)  **My HMO never recommended it** (13 %)  **I didn’t know it existed** (25%)  **I get all the support I need from other sources** (32%) |
| Plass & Koch (2001) | Aims to identify factors contributing to the participation of oncology outpatients in psychosocial support. | Quantitative  Cross-sectional survey | 132 patients in four oncology outpatients’ clinics in Hamburg.  95 (72%) had not attended psychosocial support. | Psychosocial support, oncological outpatient clinics  Free, psychosocial and somatic care is financed by health insurance organizations as part of cancer treatment in Germany  Germany | **Feeling sufficient supported by family and friends**. Most frequent source of support was family (n=75), followed by friends (n=54).  **Preferring to talk with someone else.** Prefer to talk with their doctor (n=43)**.**  Prefer to talk with their clergyman (n=10)**.**  **Not in need of assistance.** Find that they can cope on their own (n=43), having no emotional problems (n=33), Other patients were more in need of help (n=24)  * Multiple responses were possible |
| Ussher, JM, et al (2008) | Aims to identify factors influencing participation and long-term retention in cancer support groups. | Mixed method  Open ended questionnaire (conducted with dropouts)  Individual or focus group interview (conducted with non-attendance) | 87 people who had dropped out of 46 cancer support groups out of which 71 was people with cancer  27 individuals who have never attended. | Peer support groups  Free, available to all diagnosed with cancer.  Australia | *Dropouts’ reasons:*  **Practical**: Transport problems, timing of meetings, clashes with other commitments, physical health  **Time to move on:** Improved health condition made support group irrelevant  **Dissatisfaction with the group**: Unprofessional or dominant leader, depressing, mismatch between individual and the group  *Non-attendance reasons*:  **Resisting position as cancer patient**: not self-identifying as a person with cancer  **Personality factors:** **groups are not for me**: Not normal to ‘open-up for people’ or being ‘introvert’, able to cope by themselves, to distressed to let people know.  **I currently have enough support**: Existing support network seen in the form of hospital treatment, relatives or religion.  **Support groups are negative places**: Fear of bad stories and death would influence wellbeing.  **Lack of knowledge**: Many did not have knowledge or understanding of what support groups entailed.  **Finding the right group: Wanting “people like me”:** Experiences of not being able to relate to people attending such groups.  **Practical issues**: Timing, conflicts with commitments and transport distance. |
| Fitzpatrick TR & Remmer J (2011) | Aims to examine the relationship between needs, expectations, and attendance patterns among users of a cancer wellness center. | Mixed Methods  Randomly design  Telephone interview using a brief questionnaire | 33 people living with cancer not participating in rehabilitation.  16 was registered to cancer wellness center program but never attended  17 attended three times or less. | Varied exercise programs  Art studio  Support groups  Cooking and nutrition  Music and art therapy  Free, financed through Canadas insurance program  Canada | **Health and illness**: fatigue from chemotherapy, pain and physical limitations.  **Other Barriers:** parking issues, demanding work-schedule, childcare, distance from center, not fitting preferences, to long registration process, lack of service to specific patient group, not directed to their specific needs. |
| Clover et al (2015) | Aims to explore the reason why cancer patients who experience distress do not seek help. | Quantitative  Cross-sectional survey | 311 oncological patients at a regional cancer center, who scored 4 or more on the Distress Thermometer.  221 (71%) declined help. | General offer of psychosocial support targeting cancer patients.  Free, available to all diagnosed with cancer  Australia | **I prefer to manage myself** (n=99, 46%)  Authors states that it is unclear what underlies this preference. Including if it’s a genuine preference to handle issues themselves or if it reflects an underlying reluctance to seek help and what kind of strategies is used to manage distress.  **Getting help from another source** (n=52, 24%)  **Rates distress not severe enough to seek help** (n=50, 23%)  People indicating this reason had significant lower score on the Distress Thermometer and on PSYCH-& for clinical anxiety & depression. |
| Handberg et al (2015) | Aims to describe male cancer survivors’ barriers toward participation in cancer rehabilitation. | Qualitative  5-month ethnographic fieldwork  Participant observation  Interview. | 35 male cancer survivors being treated in three hospital wards | General Rehabilitation program  Free, available to all diagnosed with cancer as part of the Danish national health system  Denmark | **Fear of losing control**:  Rehabilitation was associated with reduced manliness, and many found it difficult to relate to sentimental or tears. Support was synonym with weakness.  The men disassociate with the sympathy and dependency support symbolizes for them.  Confrontation with dead and other sick people spiked fear and threatened the sense of control.  **Striving for normality**:  Believe that rehabilitation would hinder autonomy and the possibility to forget and move on. Need of maintaining pre-identity outside the hospital.  Authors connect barriers on three levels:  1.Identity: Masculine ideals can not reconcile with participation in rehabilitation.  2. Socially: Fear of becoming independent of others and talk of emotions or death.  3. Existentially: want to move on and not confront with death. |
| Cheville AL, et al (2017) | Aims to describe the proportion and characteristics of patients with late-stage cancer that are and are not receptive to receiving rehabilitation programs, as well as the rationale for their level of interest. | Mixed Methods  Prospective longitudinal study.  Telephone interview using open ended questions | 311 people diagnosed with stage IIIC or IV non-small or extensive stage small cell lung cancer.  212 was not interested in rehabilitation  Participants with no interest in rehabilitation programs with a functional limitation >4/10 were asked for reasons. | General Rehabilitation program  No structural frame presented, interest in general rehabilitation  United States | **Feeling too busy**: Conflict with medical or other appointments.  **Rehabilitation is unnecessary:** Have the help they need, can manage on their own, previously received therapy or remained physically active. Most dominant were experience of being able to manage on their own (n=36),  **Need to finish treatment or test**: Waiting to recover from or finish treatment, waiting for test response.  **Rehabilitation would not be beneficial**: Experience with rehabilitation not being helpful (n=36), or experience with rehabilitation gave them the support they needed. Others did not believe rehabilitation would be beneficial.  **Participation would be burdensome**: Cancer and treatment symptoms making it too burdensome (n=29). Practical issues as transportation or affordability (n=5)  Cancer patient’s misperception of the role of rehabilitation is highlighted as a barrier to participation.  People with limited community and household mobility were often not interested in rehabilitation |
| Hardcastle et al. (2018) | Aims to investigate factors influencing non-participation in a structured exercise program for cancer survivors and to explore survivors’ experiences and attitudes in relation to physical activity participation. | Mixed methods  Interviews  Face-to-face or telephone | 153 patients were invited to participate  20 people diagnosed with cancer in previous 5 years.  9 registered programs but cancelled registration    5 failed to attend  6 dropped out during the 2 years program- | Exercise program  Specifically tailored to cancer survivors  Free, run by non-profit organization  Australia | **Availability of the program**: Program fully booked, conflicting with cancer treatment  **Access, time and cost**: Location and transportation issues, too expensive to continue exercise after program.  **Lack of motivation or confidence:** “Just being lazy” “out of habit” “lack of self-discipline”. Afraid to do exercise wrong and look stupid.  **Unwell or fatigue**: Feeling too sick. Mostly people doing treatment  **Physical activity preferences**: Exercise is not accustomed to physical conditions and abilities. Preference for homebased exercise program.  **Knowledge of physical activity guidelines:** Patients could not cite the PA-guidlines but did not explain this as a reason for nonparticipation.  **Lack of referral or advice:** Did not recall receiving advice or referral for PA. Most were aware of the program through advertisements. No were referred by their oncologist. |
| Toivonen K, et al (2020) | Aims to understand barriers to participation in and completion of mindfulness-based cancer recovery. | Mixed methods  Self-reported questionnaire  Open-ended semi- structured interviews | 46 people registered for mindfulness-program participated in the study  6 dropped out. Of those 4 participated in survey, 3 in open-ended questions and 6 got interviewed.  Completers and drop-outs reasons were pooled together. Results shows that dropouts experienced same type of barriers as the participants, rather than distinct ones – but they tend to experience the barriers more often. | Mindfulness-based cancer recovery  Group-based  Free, program available to all people diagnosed with cancer in Southern Alberta  Canada | **Practical barriers**: Conflicting with family obligations and daily life.  **Person related barriers**: Lack of motivation, self-control, concentration. Having enough social support. Dropouts found it especially difficult to prioritize rehabilitation in daily life.  **Cancer specific barriers**: Conflict with hospital appointments. Side effect from treatment.  **Program-related barriers: (**Least reported) Uncertainty if they did it ‘right’. Program did not meet expectations. Drop-out did not experience benefits. |
